# Exploring the Handler-Dog Connection within a University-Based Animal-Assisted Activity

**DOI:** 10.3390/ani9070402

**Published:** 2019-07-01

**Authors:** Stephanie Kuzara, Patricia Pendry, Nancy R. Gee

**Affiliations:** 1 Department of Human Development, Washington State University, Pullman, WA 99163, USA; 2 Department of Psychology, State University of New York, Fredonia, NY 14063, USA; 3 WALTHAM Centre for Pet Nutrition, Leicestershire LE14 4RT, UK

**Keywords:** animal visitation program, handler, dog, animal-assisted activity, university campus

## Abstract

**Simple Summary:**

There has been a tremendous increase in the use and popularity of animal assisted activities (AAAs) on university campuses around the world. Despite a recent increase in research to examine the efficacy of AAAs, little is known about how handlers and their dogs interact to facilitate student experiences during these programs. This study aims to explore how handlers interact with their dogs before and during interactions with students. It was found that handlers spent more time petting their dog, talking to their dog, and restraining their dog by the leash when students were present. Handlers also displayed distinct interaction styles toward their dogs, including hands-off, permissive, authoritative, and authoritarian styles. Understanding the nature of interactions between handlers and their dogs is important for determining what factors contribute to program quality and efficacy while safeguarding animal wellbeing.

**Abstract:**

There has been an increase in research concerning the quality of dyadic interactions between humans and dogs in university-based animal assisted activities (AAAs). While interactions between students and dogs are commonly an area of focus, studies examining interactions between handlers and their dogs are needed. We coded 10-min long video-recorded observations (*N* = 151) using a mixed methods approach to capture the duration and frequency of dog-directed handler behavior (i.e., postural state, verbal and physical contact) before and during interactions with student participants in an AAA. Analyses showed a significant decrease in the proportion of time handlers spent petting their dog, and a significant increase in the proportion of time handlers spent sitting on the floor and restraining their dog by the leash in response to student introduction. Two dimensions of handlers’ dog-directed behavior emerged (e.g., warmth, control) revealing distinct handler interaction styles: Hands-off (L Warmth, L Control), permissive (H warmth, L control), authoritative (H Warmth, H Control), and authoritarian (L Warmth, H Control). Handlers’ interaction styles were influenced by student presence, leading some handlers to increase warmth behaviors directed to their dogs, while others decreased such behaviors. Implications for the facilitation of university-based AAAs are discussed.

## 1. Introduction

Animal-assisted activities (AAAs), or activities that incorporate human animal interaction to promote student well-being, have grown increasingly popular on university campuses to combat high student stress levels [1]. While initial evidence suggests positive outcomes for student participants, including reduced perceived stress [2], positive changes in mood [3,4,5] and reductions in student cortisol levels [6], there is limited research on the nature of human animal interactions during university-based AAAs. There appears to be no research on the extent to which dyadic functioning between handler and dog is manifested during university-based AAAs, nor do we have knowledge about the extent to which dyadic functioning between handler and animal may relate to triadic contexts, such as when handlers and dog teams engage with students. In fact, virtually nothing is known about the quality of dyadic and triadic interactions during AAAs in shaping effects of university-based programs on human or animals.

Increasing our knowledge in this realm is important for several reasons. First of all, the handler-dog team usually plays a central role in the delivery of university-based AAAs. The handler is usually the dog’s owner and many organizations that register handler-dog teams require an individual to be familiar with a dog for a minimum amount of time (e.g., 6 months, 1 year) before acting as the animal’s handler [7]. Also, handlers are often regarded as the animal’s best advocate during AAAs, meaning that it is the handler’s responsibility to be attuned to their animal’s needs and act accordingly to safeguard the animal’s wellbeing [8]. Although university-based AAAs still feature many different approaches, including the use of shelter animals, faculty or student owned pets and untrained, ad-hoc handlers, implementation approaches that feature trained, evaluated and certified handler-dog teams are recommended and have become more prevalent. As such, documenting handler behavior during AAAs and understanding the handler role in shaping the quality of interaction is essential.

A second reason to increase our knowledge about the handler-dog relationship is that the quality of dyadic functioning between handler and dogs—in general and in the context of university-based AAAs—may influence the animal’s ability to cope with potential stress experienced in this setting. Although not conducted in university settings, several studies have reported behavioral indications of dog stress being influenced by the quality of the relationship with their handler. For example, one study reported that more time spent between handler and a military dog was associated with decreased observations of abnormal behaviors traditionally linked to chronic stress in dogs (e.g., “paw licking”, “destroying material”, “diarrhea”, “howling”, “pacing”, “running around after its tail”, “barking”) [9]. Another study reported that the owner’s personality affected the behavioral expression, dyadic function, and stress load of the animal via interaction style [10]. Owners who rated higher on the neuroticism scale felt more attached to their dog and showed their dog more attention, and their dogs were rated as more confident and friendly, although distant to other people when with a female owner. In contrast, owners that scored higher on the extroversion scale treated their dog as a partner to share activities with and had more friendly but agitated dogs that were rated as moderately stressed [10].

In addition to behavioral evidence, several studies suggest that the quality of handler-dog relationships can have a physiological impact on animal welfare. For example, a study by Schoberl and colleagues [11] reported that owners high in neuroticism had dogs with lower morning cortisol levels that spent more time in proximity to their owners and approached their owners more often in an experimental test. Furthermore, dogs who were classified as securely attached to their owners had lower cortisol reactivity than dogs that were classified as insecurely attached [12]. Dogs considered to be a “social partner” or a “meaningful companion” by their owners had low morning salivary cortisol control values, indicating that the quality of owner-dog relationships is reflected physiologically. Schoberl et al. [11] compare this relationship to the bond between caregiver and infant, or parent and child. Related research examined patterns of attachment styles between owners and dogs [13] and found those patterns to be similar to those of human primary caregivers and their infants. This line of research demonstrates that the relationship between the dog and owner influences the dog’s stress coping. Since humans have an ethical responsibility when using animals were our own benefit, conducting further research may help inform sound and safe implementation practices of AAAs on university campuses.

A third reason for addressing this shortcoming in the literature is that the quality of dyadic functioning between handler and dog in the AAA context may influence the student experience and mediate the efficacy of observed program effects on student outcomes. Only one study has assessed the impact of handler presence on student outcomes during a university-based AAA, indicating that, when a handler was present, students reported lower mood compared to when only the dog was present [4]. Since this study did not assess interactions between the handler and the dog and did not take into account indicators of dyadic relationship quality, further research is needed to examine these dynamics in shaping the efficacy and effectiveness of university-based AAAs in shaping student stress relief and comfort.

There is in fact, evidence in other contexts, particularly for military and detection dogs, that task performance is influenced by the strength of the handler-dog bond. For example, a study assessing the performance of a detection dog-handler team, measured by the speed at which a dog makes a positive detection, was reported to be lower when the dog was paired with an unfamiliar handler compared to when the dog was paired with a handler it was highly bonded with, suggesting that the relationship between the handler and the dog is important to team performance [14]. Last but not least, gaining this knowledge is also relevant to ensure student safety and reduce institutional liability.

In sum, more research is needed to understand how handlers and dogs interact during university-based AAAs, what behaviors are indicative of handler-dog relationship quality, and how relationship functioning in the dyad during AAAs is affected by engagement with students and vice versa.

This study describes the quality of the handler-dog relationship based on examining behavior of handlers toward their dogs during a university-based AAA. Given that there is little prior research examining handler-dog interactions in these settings, we used approaches of grounded theory and open, axial and thematic coding [15], combined with quantitative approaches to capture the nature, frequency, and duration of dog-directed handler behavior (i.e., postural state, physical and verbal contact). Next, we examined whether patterns of dog-directed handler behavior indicative of the quality of dyadic relationship functioning could be identified and assessed those patterns before and during students’ presence. This study seeks to address the following research questions:

1. What dog-directed behaviors do handlers perform in the context of a university-based AAA?
2. Do handlers display patterns of behavior and how can these patterns be characterized?
3. Do handlers’ interaction styles with their dogs differ in response to student presence?

## 2. Materials and Methods 

All protocols and surveys were approved by the Institutional Animal Care and Use Committee (IACUC Number 04785-006), the WALTHAM™ Animal Welfare and Ethical Review Board, and the Institutional Review Board (IRB Number 14918-005) at Washington State University.

### 2.1. Participants

This study involved capturing video-recordings of the behaviors of handler-dog teams during a 10-min period of a ‘meet and greet’ session (*N* = 151). This took place during a randomized controlled efficacy trial examining the effects of infusing various levels of human-animal interaction into an evidenced-based stress prevention program targeting university students (*N* = 309). Of the three groups to which students were randomly assigned, only those featuring human animal interaction were included in this study. (Note: The results on efficacy are reported elsewhere)**.**

Handler-dog teams were recruited from Palouse Paws, a regional community partner of the Pet Partners national organization. Participating teams consisted of 18 male, neutered dogs and 11 female, spayed dogs (*M*_age_ = 4.4 years, *Age*_min_ = 1 year, *Age*_max_ = 12 years, *SD* = 3.4). More than half the dogs were either Labrador Retrievers or Golden Retrievers (*n* = 16) (*n*_other_ = 13). Handlers (*N* = 28, *M*_age_ = 42.3 years, *Age*_min_ = 18 years, *Age*_max_ = 70 years, *SD* = 18.5) were mostly female (*n* = 24) and had an average of 2.79 years of experience in volunteer animal-assisted work (*SD* = 1.69 years). On average, teams participated in 4 sessions (*session*_min_ = 1, *session*_max_ = 15, *SD* = 3 sessions).

All dogs were trained and passed evaluation to obtain a Pet Partners registration, which assesses the dogs’ reaction to various types of handling and potentially fear-producing stimuli. Dogs were required to demonstrate friendly body language, acceptance of human interaction in a pleasant matter, and minimal distress signals. Each dog had to be physically healthy and up to date on vaccinations. According to Pet Partners regulations, all handlers must know the animal for at least 6 months before they are able to take an evaluation to become a registered team. Only one handler in the current study was not the direct owner of their dog but had known the dog for greater than 6 months and had passed a Pet Partners evaluation with that dog. Finally, dogs could not be on a raw protein diet and must be bathed and groomed within 24 h of AAA participation. In this study, each dog team attended an informational meeting prior to participating, in which the study background and procedures were explained in detail.

### 2.2. Procedure

Handler-dog teams attended four, 1-h long sessions in which they interacted with a total of 212 undergraduate students assigned to conditions with human-animal interaction over a period of 4 semesters. Each handler-dog team was assigned to one of 7 individual stations for the duration of each session with free access to a water bowl. Sony HandyCam cameras were positioned on tripods at each station and angled to capture handler and dog behavior. Additional cameras were arranged at stations in the rare occasion in which there was more than one dog team at a station. Some cameras were outfitted with a shotgun microphone and windscreen to reduce obstructions to sound quality. Camera view was monitored at each station throughout programming to ensure clear visuals of dog and handler and were repositioned as needed. Handler-dog teams commonly arrived 10–15 min before program sessions started, allowing teams to get settled into their stations before students entered the room. Students waited for permission to enter the room out of sight of animals. Once they did enter, groups of 4–5 students were instructed to approach a team of their choosing but to do so while minimizing crowding of the animals and avoiding blocking the cameras. Students were not given any instructions about how to interact with animals or handlers.

### 2.3. Analytic Approach and Rationale

Coding was conducted on the first 10 min of each video session and included the 5-min prior to student arrival, in which handlers were ‘alone’ with their dogs at their station, and 5-min during student interaction, in which students first approached and interacted with the handler-dog team during a ‘meet and greet’ activity. Behavior was first assessed during the entire 10 min to determine what dog-directed behaviors handlers perform in the context of a university-based AAA, thereby addressing our first research question. Behaviors were coded manually using the observational analysis software, Mangold INTERACT (Mangold International, Arnstorf, Germany). An ethogram was designed through the use of Strauss and Corbin’s [15] grounded theory approach to first determine behaviors that might be prevalent and relevant in this unique context. While grounded theory largely encourages an open and fresh perspective when exploring new concepts, Strauss and Corbin encourage engagement with the literature as a means of identifying what is important to the developing concept [15]. Therefore, the literature was also consulted to determine behaviors that have been coded in related studies, including proximity and physical contact [16,17].

Open coding was conducted by recording all dog-directed behaviors displayed by handlers that were observed during the 10-min video. This process was conducted until a thorough list of behaviors were accumulated and no new dog-directed behaviors arose. Next, axial coding was conducted to determine relationships between the behaviors identified during open coding. Initial categories of handler behavior were created, including physical contact directed to the dog (i.e., petting, restraining the dog by the body, by the leash, or by the collar, grooming, kissing, and physical commands), handler postural state (i.e., sitting on a chair, sitting on the floor, walking, or standing), and handler verbalizations directed towards the dog were coded (i.e., verbal commands, praise, corrections, and neutral talking).

Behaviors were coded as either duration or frequency depending on the nature of the behavior. Behaviors that occurred as brief events, such as when the handler gave their dog a command, were coded as frequency, whereas behaviors that occurred over a longer period of time, such as standing, were coded as duration. The duration of time in which the handler was not visible in the camera frame was coded as out of view and substracted from the final video duration. The proportion of time spent performing a duration behavior was calculated by dividing the number of seconds performing the behavior out of the total duration of visible time. Similarly, the frequency of a behavior was divided out of the total duration of visible time to calculate the number of times a behavior was performed per visible minute. Behaviors were coded by 5 trained coders and reliability was assessed at regular intervals. Handler postural state had a final reliability score of 0.92, while handler physical contact had a reliability score of 0.84, and handler verbal contact had a reliability score of 0.87.

To determine if there was a difference in the duration or frequency of handler behaviors before and during student interaction, we conducted Wilcoxon signed-rank tests. Since assumptions of normality and homonogeneity of variance of difference were not met, this non-parametric test was used specifically to consider the dependency within samples. Normal approximation was used to calculate effect sizes using the Wilcoxon statistic (W_+_, the sum of the ranks of the positive values) since the sample size of each group was greater than 10 [18]. According to Mann and Whitney [19], normal approximation has been acknowledged as appropriate for small sample sizes, as the distribution of the Wilcoxon statistic approaches normality even in samples sizes as low as 8. Effect size is reported as a common language effect size statistic (r), or the proportion of cases (matched pairs) in which the percent of behavior is greater during student interaction compared to percent of behavior before student interaction. This statistic was calculated by dividing the number of positive ranks out of the total number of pairs. Ties (i.e., duration was equal before student interaction and during student interaction) were removed from the total.

During axial coding, we also noticed distinct differences in the valence of handlers’ dog-directed interactions, which appeared to reflect an underlying motivation of the behavior. We assessed behaviors for commonalities beyond just the type of interaction (i.e., physical, postural, verbal), which led to the emergence of two dimensions: Handler warmth and handler control. Through the grounded theory process, it became evident that there were distinct patterns to handlers’ interaction styles characterized by these two dimensions. We noted that some handlers predominantly displayed warmth behaviors when interacting with their dogs, while others mostly engaged in behavior exerting control. Additionally, we recognized that warmth and control were not mutually exclusive. Rather, handlers used a combination of warmth behavior and control behaviors when interacting with their dogs. As mentioned previously, grounded theory encourages engagement with existing literature as a means of identifying what is important to the developing concept [15]. The emergence of themes of warmth and control led us to consult the parenting literature, which has incorporated these dimensions to identify distinct parenting styles [20]. The dimensions described in that literature appear to constitute a useful framework for coding and analyses.

Handler behavior was thus identified according to these dimensions and behaviors were grouped accordingly. Petting, grooming, praising the dog, talking to the dog, and treating the dog were categorized as behaviors reflecting warmth. Kissing the dog was initially included as a behavior indicative of warmth, but kissing incidences were extremely rare and not included in composite scores. Restraining the dog, giving the dog a command (physical or verbal), and giving the dog a verbal correction were identified as indicative of control. Next, we examined the distributions of each behavior with the intent of assigning each behavior a standardized score for composite score calculation. Given non-normality and positive skewness of the data, we transformed the data using square-root transformations as suggested by Howell [21]. Z-scores were then calculated for all transformed warmth and control behaviors individually from which composite scores of handler warmth and control were calculated at each time point (e.g., before and during student interaction) by averaging z-scored behavior within each dimension. These composites were then used to plot each handler’s warmth and control scores (positive X negative; negative X positive; negative X negative; positive X postive) in a 2 × 2 model to determine each handler’s interaction style before and during student interaction.

## 3. Results

### 3.1. Handler Postural State, Physical Contact Behavior and Verbal Communication 

On average, trimmed video segments representing time before student approach were 293.0 s in duration (*SD* = 25.9 s, *M* total visible time = 255.0 s, *SD*_visible_ = 88.2 s). Trimmed videos representing time during student interaction were 310.0 s in duration (*SD* = 22.5 s, *M* total visible time = 308.4 s). The most common postural state performed by handlers over the total 10 min was sitting in a chair at 67.0% of the total visible time (*SD* = 0.40). The second most common postural state was sitting on the floor, with handlers spending 27.5% of total visible time sitting on the floor (*SD* = 0.39). Handlers spent a smaller portion of time standing (3.4%, *SD* = 0.10) and walking (0.6%, *SD* = 0.01).

The most common physical contact behavior directed from the handler to their dog was petting at 12.0% of total visible time (*SD* = 0.10). Handlers did not engage in physical contact with their dogs (i.e., neutral) for the majority of visible time (83.0%; *SD* = 0.20). Other physical contact behaviors directed from the handler to their dog were less frequent. Handlers restrained their dog’s body, on average, 0.02 times per minute, or 2 times across the 10 min observation period (*SD* = 0.07). Handlers restrained their dog by the collar, on average, 0.01 times per minute, or 1 time across the 10 min observation period (*SD* = 0.01). Handlers restrained their dog by the leash, on average, 0.02 times per minute, or 2 time across the 10 min observation period (*SD* = 0.03). Handlers gave their dog, on average, 0.2 physical commands per minute, or 2 commands across the 10 min observation period (*SD* = 0.30). Incidences of the handler rewarding their dog with food (0.07 treats given per minute; *SD* = 0.20) or kissing their dog (0.02 kisses per minute, *SD* = 0.10) were rare.

The most common verbal communication behavior exhibited by handlers was talking, with 3.0% of visible time spent talking to their dog (*SD* = 0.03). Handlers also gave an average of 0.24 verbal commands to their dog per minute, or a total of 2.4 verbal commands across the total 10-min period (*SD* = 0.30), and 0.19 verbal corrections per minute, or a total of 1.9 corrections across the total 10-min period (*SD* = 0.20). Handlers gave their dog, on average, 0.20 non-verbal commands per minute, or 2 commands across the 10-min observation period (*SD* = 0.30).

#### Changes in Handlers’ Dog-Directed Behavior in Response to Student Approach

**Figure 1:**
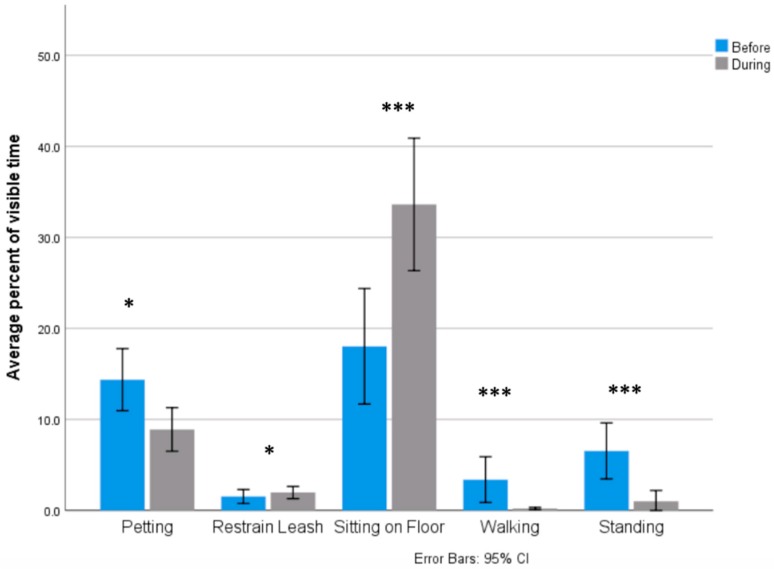
Significant increases in percent visible time spent displaying several behaviors before and during student interaction. * Significant at the 0.05 level. ** Significant at the 0.01 level *** Significant at the 0.001 level.

Handler postural state varied significantly in the 5-min section prior to student approach and the 5-min segment following student approach (Figure 1). Wilcoxon signed-rank tests revealed a statistically significant increase in percent visible time spent sitting on the floor (*M*_before_ = 0.18, *M*_during_ = 0.34, *p <* 0.001, *r* = 0.86) following student approach. Thus, handlers increased the amount of time spent sitting on the floor, thereby increasing their proximity to both their dog and the students. According to the common language effect size, there is a 86% chance that the percent visible time spent sitting of the floor was greater during student interaction compared to before student interaction if a handler observation was randomly selected. Additionally, Wilcoxon signed-rank tests revealed a statistically significant decrease in percent visible time spent walking following student approach (*M*_before_ = 0.03, *SD* = 0.16, *M*_during_ = 0.00, *SD* = 0.01, *p* < 0.001, *r* = 0.22) and a statistically significant decrease in percent visible time spent standing following student approach (*M*_before_ = 0.07, *SD* = 0.19, *M*_during_ = 0.01, *SD* = 0.07, *p* < 0.001, *r* = 0.73). Therefore, if a handler observation was randomly selected, there was a 22% chance that the percent visible time spent walking was greater before student interaction compared to during student interaction, and a 73% chance that the percent visible time spent standing was greater before student interaction compared to during student interaction.

Several physical contact behaviors varied significantly in the 5-min section prior to student approach and the 5-min segment following student approach. Wilcoxon signed-rank tests revealed a statistically significant decrease in percent visible time spent petting their dog following student approach (*M*_before_ = 0.14, *SD* = 0.21, *M*_during_ = 0.09, *SD* = 0.15, *p* = 0.048, *r* = 0.46). Therefore, if a handler observation was randomly selected, there was a 46% chance that the percent visible time spent petting the dog was greater before student interaction compared to during student interaction. Additionally, there was a statistically significant increase in percent visible time spent restraining their dog using the leash (*M*_before_ = 0.015, *SD* = 0.05, *M*_during_ = 0.020, *SD* = 0.04, *p* = 0.006, *r* = 0.65) (Figure 1). Therefore, if a handler observation was randomly selected, there was a 65% chance that the percent visible time spent restraining the dog using the leash was greater during student interaction compared to before student interaction.

### 3.2. Handler Interaction Style: Warmth and Control 

#### Thematic Results

**Figure 2:**
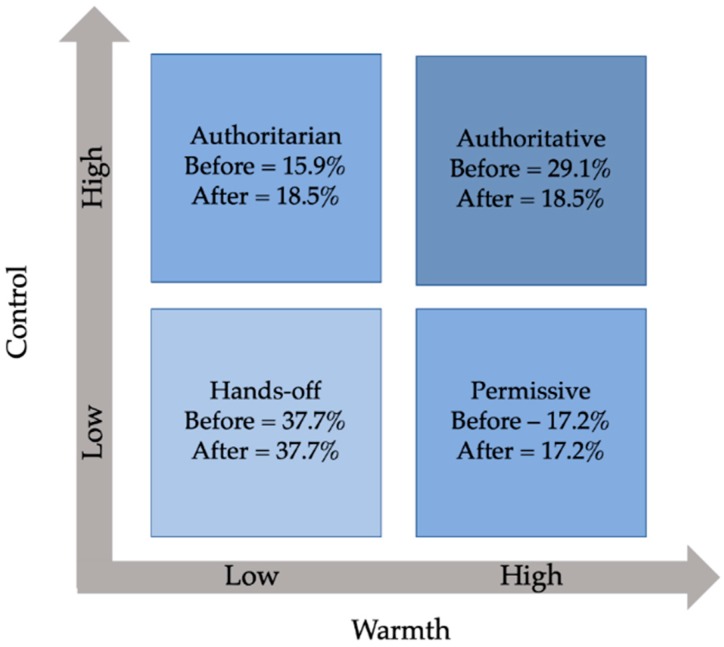
Dimensions of dog-directed interaction behavior by handlers.

As described in the analytic approach section, during open and axial coding, differences in the valence of handlers’ dog-directed behavior emerged along two dimensions (i.e., warmth and control), which when examined simultaneously in a 2 × 2 model resulted in the following handler interaction styles (Figure 2). Handlers who displayed high warmth (i.e., a positive Z-score for handler warmth) and high control (i.e., a positive Z-score for handler control) were classified as authoritative handlers. These handlers displayed a combination of warmth and control behaviors when interacting with their dogs. For example, an authoritative handler may frequently initiate physical and/or verbal commands asking their dog to sit or lay down, followed by praise and/or petting behavior when the dog complies. Handlers who displayed high warmth (i.e., a positive Z-score for handler warmth) and low control (i.e., a negative Z-score for handler control) were classified as permissive handlers. A permissive handler may engage frequently in petting behavior, praise, and/or talking to their dog, while refraining from directing commands or restraining their dog’s movement. Handlers who displayed low warmth (i.e., a negative Z-score for handler warmth) and high control (i.e., a positive Z-score for handler control) were classified as authoritarian handlers. An authoritarian handler may frequently direct physical and/or verbal commands toward their dog without engaging in a high level of petting behavior and rare instances of praise even in response to compliance. Finally, handlers who displayed low warmth (i.e., a negative Z-score for handler warmth) and low control (i.e., a negative Z-score for handler control) were classified as hands-off handlers. A hands-off handler may be engaged with other handlers and/or materials at this time or may be sitting quietly without interacting with their dog. Interaction styles were determined before and during student interaction. Interpretations of these styles and changes therein upon student introduction will be described next.

We found that a hands-off interaction style was the most common handler interaction style prior to student interaction (37.7%). The next most common interaction style before students were present was an authoritative style (29.1%), followed by a permissive style (17.2%) and an authoritarian style (15.9%). This pattern shifts when students entered, with the percentage of handlers displaying an authoritative style decreasing (26.5%), and the percentage of handlers displaying an authoritarian style increasing (18.5%). Interestingly, the percentage of handlers displaying a hands-off style or a permissive style did not change when students were present.

As handler-dog teams participated in more than 1 session (*session*_mean_ = 4 sessions; *session*_min_ = 1, *session*_max_ = 15), the consistency in which handlers displayed the same style across all videos in which they appeared was also determined. The majority of handlers (78.5%) displayed the same style before students were present in the majority (50% or greater) of all videos in which they appeared. When students were present, only 67.9% of handlers displayed the same style across the majority of videos in which they appeared. Therefore, handlers adapted their style when interacting with their dog in the presence of students.

The degree to which handlers changed their interaction style when students approached was determined by assessing what style was displayed prior to student approach and which style was displayed when students were present. Of the handlers who displayed a hands-off style before students approached, 44% retained a hands-off style when students were present. The most commonly seen shift in interaction style was from a permissive style before students were present to a hands-off style when students were present (42%) indicating that handlers decreased the degree to which they interacted with their dog when students were present, likely to allow students to interact with the dog instead. However, this was closely followed by a shift from an authoritarian style to an authoritative style when students were present (36%), suggesting that handlers who initially displayed an authoritarian style, increased warmth behaviors toward their dog when students were present.

## 4. Discussion

This study examined how the connection between handler and dog is manifested in a university-based AAA by coding for handlers’ dog-directed behavior and identifying clear dimensions of behavior from which four distinct interaction styles emerged; hands-off, permissive, authoritarian, and authoritative. We also examined changes in handler behavior and styles in response to student introductions and observed an increase in the proportion of time spent petting and praising their dog, an increase in the proportion of time spent sitting close to the dog on the floor, increased talking to the dog, and an increase in the proportion of time spent restraining the dog’s movement with the leash. These results indicate that handlers displayed clear dog-directed interaction patterns during AAA programming, with significant changes in behavior occurring during crucial programming events, such as the initial entrance of students. These results suggest that the handler-dog relationship manifests depending on the environment and handlers adopt a “working persona” when engaged in AAA.

The emergence of distinct interactional styles characterizing handler and dog relationship functioning in dyadic and triadic context contributes a novel model for examining future interactions. Currently, the only existing models that are comparable exist within the caregiver-child literature. Comparisons can be drawn between the handler interaction styles and Baumrind’s parenting style typology. According to that typology, parenting behavior was classified according to four styles (i.e., authoritative, authoritarian, permissive, and indifferent) that differ along the dimensions of responsiveness and demandingness [22,23]. It should be noted that, while similarities exist with regards to the behavioral dimensions underlying handler interaction style, we are not arguing that the handler interaction styles mirror the predictive properties of Baumrind’s parenting styles [20]. While there is evidence that parenting styles are linked to child outcomes, the same cannot yet be determined for the relationship between handler interaction style and dog and/or participant outcomes in the short or long term. In addition, the Baumrind model is thought to represent typical parenting styles over an extended period of time consistent throughout various contexts, while our findings suggest that handler-dog interaction styles may be context-specific in that they are informed by, and perhaps adaptive to, dog and student behavior.

It is important to consider the context in which handler interactional styles occur before interpreting one style as being more advantageous than another. The period of time prior to student entrance is often regarded as ‘down time’ for handlers and dogs and is often used as an opportunity to engage with the dog, get the dog settled, or socialize with other handlers and/or attend to other materials, such as cellular devices. Behaviors indicative of warmth (e.g., petting, praising) may not be necessary during this time period in order to maintain and/or promote connection between the handler and the dog. This may be particularly true depending on the dog’s behavior and level of activity. For example, a handler may display a hands-off interaction style if their dog is resting during the period of time prior to student interaction. A hands-off style may actually be more appropriate in this context and more conducive to decreasing experiences of dog stress than a more active style. Additionally, the hands-off style may be a manifestation of a strong connection formed between handler and dog through prior interactions, such that the handler does not need to intervene or manage the dog’s behavior extensively.

It appears that handlers shift their interaction style in response to the presence of students. This shift to a “working-persona” is evident in the frequent shift from an authoritarian style to an authoritative when students are present, such that the handler directs more positive, warm behaviors toward their dog. Handler’s may assume more active interaction styles to maintain the handler-dog unit when students are present or act as facilitators of human-animal interaction and advocates for their dog’s wellbeing. For example, a number of handlers responded to student presence by assuming a more hands-off style, evident by the common shift from a permissive to a hands-off style. These handlers may perceive their role to be more removed, instead allowing students to interact with the dog unperturbed. This may also be due to proximity if the dog spends more time in proximity to students and not the handler. Future research is needed to examine links between interactional styles and dog wellbeing and/or facilitation success in order to attach value judgements to these styles as is commonly done in the parenting literature, which consistently show better outcomes for parenting employing an authoritative style [20]. Considering prior findings that the presence of a handler reduced student mood [4], a more hands-off approach might be linked to better student outcomes.

Interestingly, handlers who did shift to a different interaction style when students approached did not all display the same style when students were present. Rather, the interaction style displayed during student present varied according to the style initially displayed by handlers before students were present. Some handlers responded to student approach and interaction by increasing warmth behaviors directed to their dogs (i.e., permissive style) while other handlers responded by increasing control and warmth behaviors (i.e., authoritative style). Therefore, while, overall, the percent of handlers displaying hands-off styles and permissive styles did not change and the percent of authoritarian styles increased, this does not fully capture how handlers vary their dog-directed behaviors in response to student presence. More research is needed to tease out why this differential handler response may occur. Perhaps each handler has a preferred interaction style that they revert to when facilitating interaction between their dog and strangers, much like a working persona that is only evident in the presence of necessary stimuli (e.g., students). Interaction style may also be linked to a handler’s personality traits, as has been suggested in prior studies [10], or by the dog’s behavior or personality, with a more anxious, active, or unruly dog requiring their handler to display an authoritarian interaction style.

Adaptive handler behavior may also be due to differences in individual student behaviors directed towards the dog that require the handler to respond differently. For example, students are likely to display different greeting and interaction behavior based on their personality, level of comfort with human animal interaction in general, interest in the individual dog specifically, as well as other contextual circumstances such as the behavior of other students’ present during the small group interaction. In addition, it is likely that the dog may respond to individual students differently depending on the student’s greeting style, characteristics of the student (e.g., their size, voice level, demeanor, odor, the presence of bookbags or hats, etc.), as well as the overall dynamic in the small group setting. Handlers’ behavior is likely to vary depending on their interpretation of which facilitation behavior is most efficacious to enhance human animal interaction while advocating for their dog’s safety and wellbeing. Furthermore, the handler may shift their interaction style to better match the behavior of students within the group. For example, a handler may increase the extent to which they talk to their dog if students are relatively disengaged, or the handler may give their dog more commands to encourage engagement with less active students. On the other hand, the handler may increase warmth behaviors to calm the dog and compensate for overly enthusiastic students that may be overwhelming to the dog or encouraging the dog to become too stimulated for the context.

Further research is needed to explore the influence of changing handler interaction style based on the dogs’ handler or student-directed behavior. Handlers may display more warmth behaviors (e.g., petting, praise) toward their dog when students are present as a means of providing their dog comfort in light of the sudden environmental shift when students enter the room. Prior studies have identified social interaction and novelty as being energizing or potentially stressful for dogs [24]. Therefore, the increase in handler warmth may serve to offset this effect in such a way as to comfort and reassure their dog, but also to role-model appropriate dog-directed behaviors to the students, which may have the down-stream effect of making the situation less stressful for their dogs. There was also a significant increase in several control behaviors, including restraining the dog by the leash and giving the dog a verbal command. These behaviors may serve as a means of facilitating interaction between the handler-dog team and approaching students. Handlers often gave their dogs a command to sit or stay as student approached in order to encourage calm behavior. This is in line with the registered therapy dog guidelines, in which dogs must remain calm when greeting a strange person or dog [8]. Further research is needed to determine whether or not these behaviors actually reduce dog stress or if they potentially contribute to the already stimulating, and perhaps overwhelming, experience for the animal. Furthermore, the degree to which these behaviors serve to facilitate interaction needs to be explored. The observed increase in time spent sitting on the ground, talking to the dog, and petting the dog may represent efforts by the handler to facilitate interaction, or appropriate interaction, between the dog and the students. There is evidence that student greeting behavior influences dog behavior [25]; thus, the handler may adapt their interaction style in response to interactions between their dog and students.

The quality of the connection between the handler and their dog is likely to influence the team’s success as a calming presence during university-based AAA aimed at decreasing student stress levels and increasing well-being. While studies exploring the relationship of handler and dog in other contexts can evaluate team success through performance measures such as detection speed of search dog teams [26], or correct hits from scent detection teams [27], there is not currently a standard for evaluating or operationalizing AAA team performance or success. This study adds to the literature by providing a basis for understanding how handlers behave during programming and how they tailor their behavior to the environment, to their dog, and to program participants.

### Strengths and Limitations

A major strength of this study is the use of a grounded theory approach to identify and assess behaviors that are grounded in the data and therefore reflect reality. In addition, the study occurred in the context of a real-life efficacy trial that reflects common features of university-based AAA settings that are popular on a number of campuses across the country and as such has ecological validity. This study provides a well-supported insight into the role of handlers and how they connect with their dogs during real-life AAA conditions. The resulting framework may thus prove useful for future examinations of handler-dog interactions in these settings.

A limitation of this study is the homogeneity of the sample of handlers regarding their demographics and backgrounds. This limits the degree to which findings can be generalized across AAA populations even though our participants may seem highly representative of the typical demographic of volunteer handlers in AAA. Furthermore, this study was limited in its ability to assess the degree to which the display of any given interaction style was appropriate or effective within the given moment. For example, a hands-off handler may be responding appropriately to a resting dog; however, this value judgment cannot be extracted from our current quantitative findings. Additionally, we are unable to extrapolate to what extent interaction styles indicate the quality of the handler-dog connection. For example, a handler may continuously display a hands-off interaction style because they are highly in tune with their dog and do not need to engage in control or warmth behaviors to maintain a high level of connection. Future research can assess this question by assigning interactions a global code for handler-dog connection. And finally, this study is limited by the fact that some individuals who conducted coding were not blind to the research hypotheses and may have unintentionally interjected bias [28].

Future research should examine how dogs respond to different handler interaction styles and how dog behavior influences which style handlers display. Additionally, future research should consider assessing handler and dog behavior after student interaction, as well as during a non-therapeutic environment, for comparison. Furthermore, the connection between dyadic behavior and dog stress is needed to assess animal welfare during AAAs. More research is required to fully explore triadic interactions, or interactions between students, handlers, and dogs. Particularly, the link between handler-dog relationship and student physiological and psychological outcomes must be assessed. This will increase our understanding of how best to operationalize handler-dog team success within AAAs.

## 5. Conclusions

The current study lays the foundation for recognizing and exploring the important role the handler plays in establishing the atmosphere of AAAs. Furthermore, this study provides initial insights into how handler and dog relationships are manifested during an AAA, and how handlers appear to adapt their interaction style and dog-directed behaviors in response to changes in their environment.
